# Chemical Basis for Determining the Allelopathic Potential of Invasive Plant Wall Barley (*Hordeum murinum* L. subsp. *murinum*)

**DOI:** 10.3390/molecules29102365

**Published:** 2024-05-17

**Authors:** Beata Barabasz-Krasny, Agnieszka Tatoj, Marek Chyc, Wojciech Gruszka, Peiman Zandi, Alina Stachurska-Swakoń

**Affiliations:** 1 Department of Botany, Institute of Biology and Earth Science, University of the National Education Commission, Podchorążych 2 St., 30-084 Kraków, Poland; beata.barabasz-krasny@up.krakow.pl (B.B.-K.); agnieszka.tatoj@doktorant.up.krakow.pl (A.T.); 2 Department of Environmental Protection, Faculty of Mathematical and Natural Sciences, University of Applied Sciences in Tarnów, Mickiewicza 8 St., 33-100 Tarnów, Poland; mrsch7@gmail.com; 3 Department of Biological Sciences, Faculty of Physical Culture in Gorzów Wlkp., Poznan University of Physical Education, Estkowskiego 13 St., 66-400 Gorzów Wielkopolski, Poland; w.gruszka@awf-gorzow.edu.pl; 4 International Faculty of Applied Technology, Yibin University, Yibin 644000, China; z_rice_b@yahoo.com; 5 Institute of Botany, Faculty of Biology, Jagiellonian University, Gronostajowa 3 St., 30-387 Kraków, Poland

**Keywords:** allelopathy, false barley, *Hordeum murinum*, invasive plant, novel weapons hypothesis, phytochemical screening, secondary metabolites, stress factors, volatile metabolites

## Abstract

The study investigated compounds present in the invasive grass *Hordeum murinum* L. subsp. *murinum* and tested the allelopathic potential of this plant against common meadow species *Festuca rubra* L. and *Trifolium repens* L. Gas chromatography–mass spectrometry (GC–MS) performed separately on the ears and stalks with leaves of wall barley revealed 32 compounds, including secondary metabolites, that may play an important role in allelopathy. Two compounds, N-butylbenzenesulfonamide (NBBS) and diphenylsulfone (DDS), were described for the first time for wall barley and the Poaceae family. The presence of 6,10,14-trimethylpentadecan-2-one (TMP) has also been documented. Aqueous extracts of *H. murinum* organs (ears and stalks with leaves) at concentrations of 2.5%, 5%, and 7.5% were used to evaluate its allelopathic potential. Compared to the control, all extracts inhibited germination and early growth stages of meadow species. The inhibitory effect was strongest at the highest concentration for both the underground and aboveground parts of the seedlings of the meadow species tested. Comparing the allelopathic effect, *Trifolium repens* proved to be more sensitive. In light of the results of the study, the removal of wall barley biomass appears to be important for the restoration of habitats where this species occurs due to its allelopathic potential.

## 1. Introduction

Environmental stress factors affect biochemical, physiological, and morphological changes in plants. They impede plant growth and development, disrupt the integrity of cells, tissues or organs, interfere with hormonal regulation, block the transport of water, nutrients and organic compounds, and cause oxidative stress [1]. These threats arise from the interaction of abiotic and biotic factors where biotic stressors can be other plants, animals, bacteria, fungi, and viruses [2]. By secreting chemical compounds, secondary metabolites or substances from tissue breakdown, plants can affect the growth and development of other plants; such compounds are called allelochemicals [3,4,5] and this interaction is known as allelopathic stress [6,7]. Allelopathy is a phenomenon that can either constrain or stimulate the growth of other plants and repel predatory insects [8,9,10]. Inhibition of the growth of one species by another leads to the dominance of species with high allelopathic potential and displacement of more susceptible plants. Therefore, the phenomenon of allopathy could play significant role in ecological succession, because it supports plants’ encroachment and presence in a new habitat [11,12]. Native plant species are often more susceptible to allelochemicals from alien colonizers. It is believed that this mechanism plays a crucial role in biological invasions [13]. The novel weapon hypothesis posits that the invasive introduced species produce novel allelopathic compounds to which the native species are not adapted [14].

One species that has spread to most areas of the temperate zone world from the Mediterranean, becoming a troublesome weed, is wall barley, *Hordeum murinum* L. [15,16]. This representative of the Poaceae family is invasive in the western part of the United States, South America, Australia, and New Zealand, where it mainly colonizes grasslands and semi-deserts [17]. It is often a pioneer species in new areas, because it grows in synanthropic sites devoid of natural vegetation. The area of its occurrence is constantly expanding, mainly as a result of human activity. The species owes its expansion, especially in urban conditions, to its rapid maturity and self-pollination as well as the production of a large number of grains with hooked hairs that facilitate transport by humans and animals (epizoochory). Yield can take place over a long growing season and even on dwarf shoots. Grains germinate fast and can sprout immediately after maturity, further increasing their competitiveness [15]. The species prefers soils rich in nitrogen and phosphorus, but also grows in poor, sandy habitats, and is also found on saline soils [15,18]. It is considered a thermophilic species in urban areas, growing on dry and fresh substrates [19].

There are three subspecies of *H. murinum* [20] that differ primarily due to their chromosome numbers [21,22,23], spikelet morphology, and geographical distribution [24,25,26]. *H. murinum* subsp. *glaucum* (Steud.) Tzvelev occurs in warmer climates of the Mediterranean, while subsp. *murinum* and subsp. *leporinum* (Link) Arcang are native to the Mediterranean near continental, oceanic, and colder climates. In subsp. *leporinum* and subsp. *glaucum,* the central fertile floret is distinctly stalked. The rachis segment of the latter is considerably shorter than that of the former. The coloured rachilla extension of the lateral florets is also clearly visible in these two subspecies. In subsp. *leporinum*, this extension is linear and long, whereas in subsp. *glaucum* it is shorter and usually darker as well. *H. murinum* subsp. *glaucum* is diploid and subsp. *murinum* is tetraploid. Cytotypes of tetraploid and hexaploid were found in subsp. *leporinum* [27]. In Poland, the low variability of morphological features allows only minor geographical differentiation of the population to be distinguished [28].

Most recently, *H. murinum* expansion has been observed in urban areas [16,29], where it can survive for a long time, especially on the edges of parks and fences [30]. In this type of habitat, it is increasingly possible to observe relatively large patches where this species is the dominant species. There are no studies to date showing its allelopathic effects on common meadows plants growing also in urban greenery. The chemical composition of this taxon is also relatively poorly known. Hence, there is a clear need to conduct experiments in this topic area.

Our study aims to determine whether the expansion of *Hordeum murinum* L. subsp. *murinum* may constitute a threat to native meadow plant species by generating allelopathic stress, resulting from the chemical composition of this species. We posed the following detailed questions: (i) what chemical compounds are produced by *Hordeum murinum*, and (ii) whether aqueous extracts from *Hordeum murinum* affect the germination and early growth stages of common meadow plants. We separately tested the allelopathic potential of ears and stalks with leaves of wall barley on two meadow species, checking the response of the monocotyledonous species *Festuca rubra* L. (red fescue) and the dicotyledonous species *Trifolium repens* L. (white clover).

## 2. Results

### 2.1. Soil Analysis

Mixed soil samples collected at the research sites showed properties of pH close to neutral, low salinity, and relatively low in nitrogen content, but rich in calcium (Table 1). The remaining bioavailable ingredients (P, K, Mg) in the analyzed samples were at an average level.

### 2.2. Gas Chromatography Coupled to Mass Spectrometry (GC–MS)

Chromatographic analysis revealed 32 chemical compounds representing five main groups: aldehydes, alkanes and alkenes, carboxylic acids and lactones, phenols and alcohols, and sterols (Table 2).

**Figure 1:**
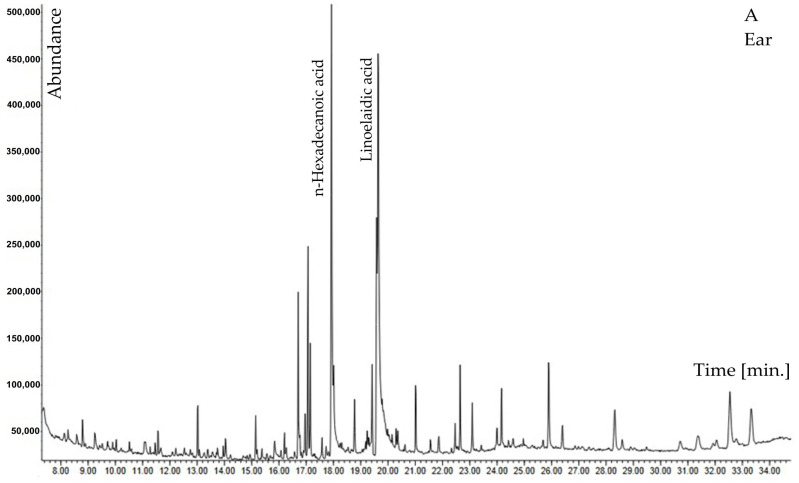

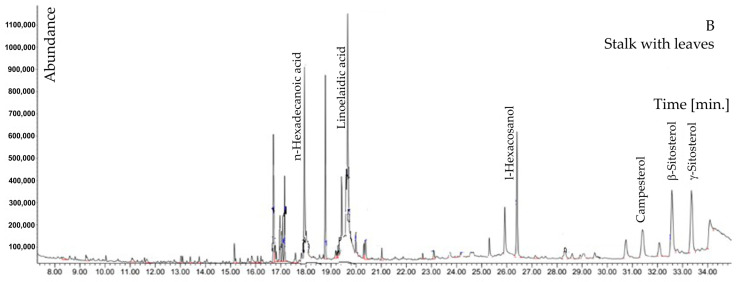
GC–MS chromatogram of methanolic extract of *Hordeum murinum* L. subsp. *murinum*: (**A**)—ears, (**B**)—stalk with leaves.

Among other compounds, N-butylbenzenesulfonamide and diphenyl sulfone containing sulfur are of special interest; 6,10,14-trimethylpentadecan-2-one was also reported. In the ears biomass of *H. murinum* subsp. *murinum*, higher percentages were found for 5-hydroxyfurfural, tricosane, and carboxylic acids (benzoic acid, n-tetradecanoic acid, n-hexadecanoic acid, linoelaidic acid). The phenol compound was identified as 2,4-ditertbutylphenol. However, in stalks with leaves, a higher percentage was recorded for neophytadiene, phenols: 1-hexacosanol, phytol, and sterols: campesterol, γ-sitosterol, β-sitosterol, 3-β-ergost-5-en-3-ol (Table 2, Figure 1A,B).

When the abundance of both fractions was compared, it was concluded that, in the case of the extract obtained from ears, it was almost half the amount of the abundance of the extract from plant stalks with leaves. Considering that the extraction conditions were the same, this means that the content of compounds extracted in methanol, in this case, was 1:2 in favor of the extract from the stalk with leaves (Figure 1A,B).

### 2.3. Germination Capacity

**Figure 2:**
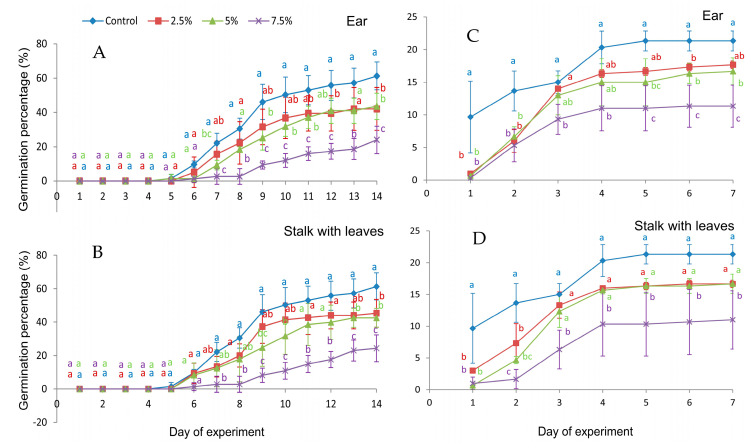
Germination percentage of seeds of *Festuca rubra* L. (**A**,**B**) and *Trifolium repens* L. cv. Grassland Huia (**C**,**D**), watered with extracts from the organs of *Hordeum murinum* L. subsp. *murinum*, with different percentage concentrations (2.5%, 5%, 7.5%). (**A**,**C**)—aqueous extracts from ears, (**B**,**D**)—aqueous extracts from stalks with leaves; mean values were calculated from 3 replicates (±SD); means marked with different letters differed significantly according to Duncan’s test *p* ≤ 0.05.

In the first 6 days of the experiment, *Festuca rubra* grains showed similar germination rates regardless of what they were watered with: extracts from wall barley ears, stalks with leaves, or the control. Starting from day 7 of the experiment, a significant reduction in GP values [%] was observed compared to the control for grains watered with 7.5% extracts from ears and stalks with leaves. However, the 5% extract of ear significantly inhibited the red fescue germination from day 7, except for days 8 and 13. Similarly, at 2.5% only on days 12 and 14, the germination was inhibited. Stalk with leaves extract at 5% significantly reduced the GP value for the red fescue grains on the 9th, 10th, 13th, and 14th day of the experiment, with a concentration of 2.5% only on the 14th day (Figure 2A,B). The germination of the white clover seeds in the first two days of the experiment was significantly inhibited by all extracts used. From the 4th day of the experiment, the stalk extracts had a significant inhibitory effect on germination only at 7.5%. Ear extracts at 7.5% also significantly weakened germination on each day of the experiment, while there were 5% extracts from the 5th day of the experiment, and 2.5% extracts only on the 6th day (Figure 2C,D).

*H. murinum* ear extracts at 2.5% and 5% significantly prolonged the average germination time of white clover seeds compared to the control, while the MGT of red fescue grains was significantly higher only when watered with 7.5% ear extract (Table 3).

Stalk with leaves extracts significantly prolonged the average germination time of white clover seeds at concentrations ≥5%, and red fescue grains at concentrations of ≥5% only. Extracts from the ear and stalk with leaves of *H. murinum* at any concentration used did not have a significant effect on the U index values of white clover seeds. However, red fescue grains watered with ear and stalk with leaves extracts at a concentration of 7.5% had significantly lower U index values; i.e., germination was more concentrated in time than in the control. The GI index values for the white clover seeds watered with each of the tested extracts were significantly lower than those of the control.

A significant reduction in the GI index of red fescue grains was caused by ear and stalk with leaves extracts at concentrations ≥5%. The average germination time of half of the white clover seeds (T_50_) was significantly longer than that of the control when watered with each of the tested concentrations of extracts from the ear of *H. murinum* and concentrations of ≥5% of extracts from the stalk with leaves. However, the T_50_ of the red fescue grains was significantly lower than that of the control when watered with ear and stalk with leaves extracts at a concentration of 7.5% (Table 3).

### 2.4. Biometric Analyses

**Figure 3:**
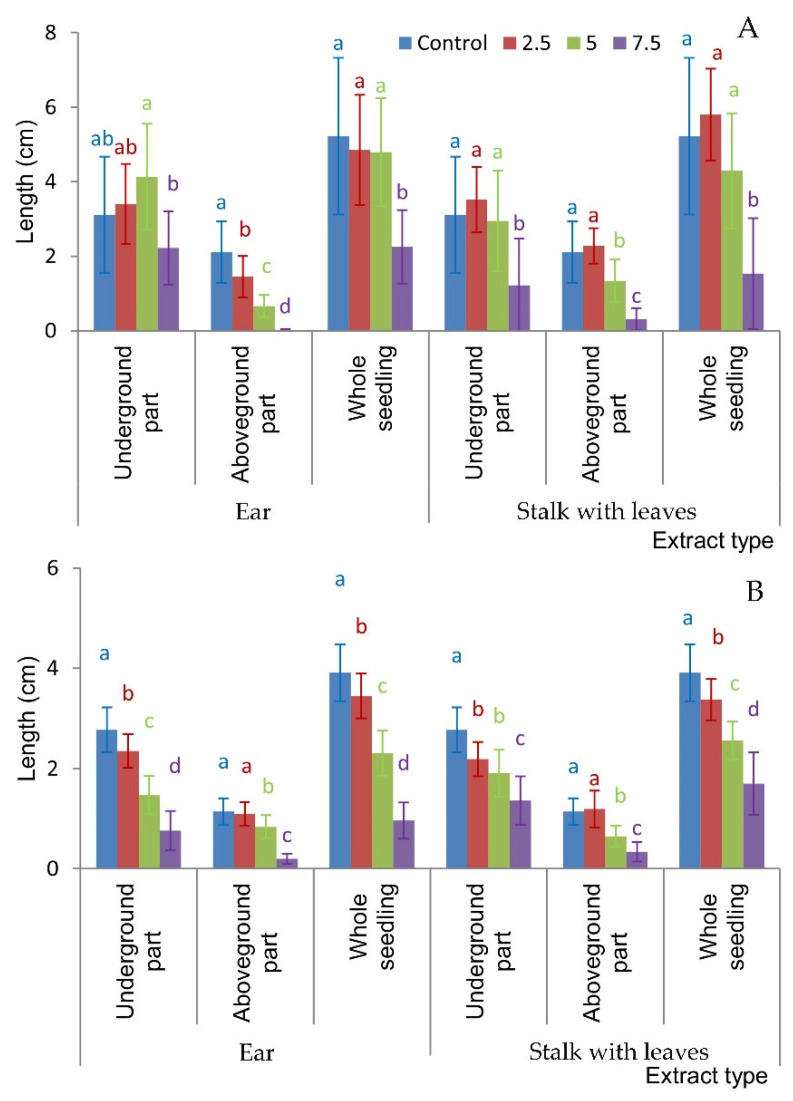
Seedling length of *Festuca rubra* L. (**A**) and *Trifolium repens* L. cv. Grassland Huia (**B**) watered with aqueous extracts of *H. murinum* subsp. *murinum* organs of various percentage concentrations (2.5%, 5%, 7.5%); mean values calculated from 10 replication (±SD); means marked with different letters differed significantly according to Duncan’s test *p* ≤ 0.05.

**Figure 4:**
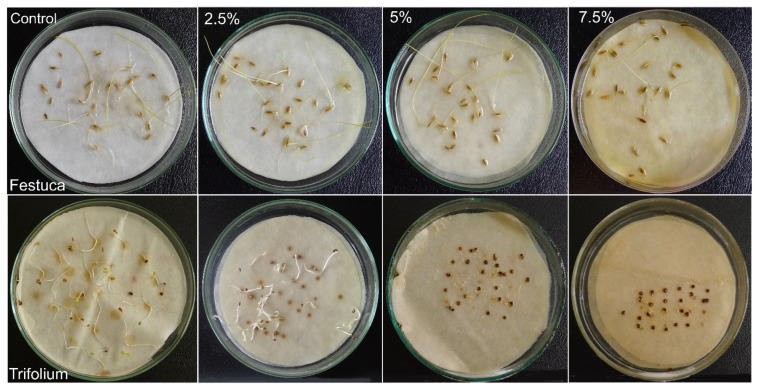
Comparison of the effect of aqueous extracts (2.5%, 5%, 7.5%) from the ears of *Hordeum murinum* L. subsp. *murinum* on the germination and early growth of *Festuca rubra* L. and *Trifolium repens* L. cv. Grassland Huia on day 7 (white clover) and day 14 (red fescue) of the Petri dish test; control with distilled water.

**Figure 5:**
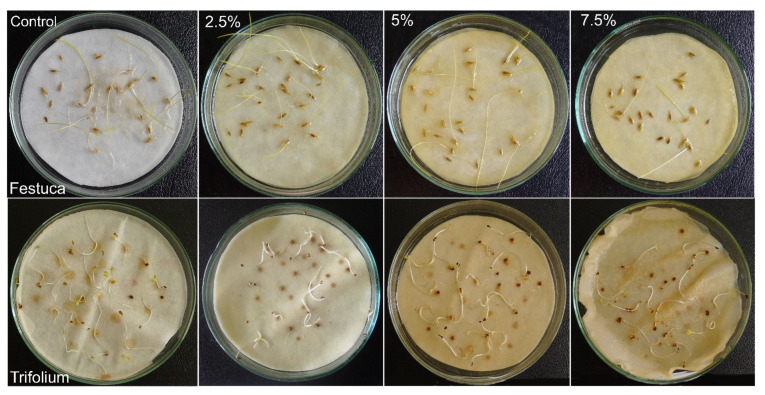
Comparison of the effect of aqueous extracts (2.5%, 5%, 7.5%) from the stalks with leaves of *Hordeum murinum* L. subsp. *murinum* on the germination and early growth of *Festuca rubra* L. and *Trifolium repens* L. cv. Grassland Huia on day 7 (white clover) and day 14 (red fescue) of the Petri dish test; control with distilled water.

The research conducted revealed differences not only in seed and grain germination but also in seedling elongation under the influence of aqueous extracts of *H. murinum* subsp. *murinum*. The elongation growth of whole *F. rubra* seedlings was significantly lower when watered with extracts from the ear and stalk with leaves at the concentration of 7.5%, but the growth of the aboveground part was significantly inhibited even with extracts from the ear at ≥2.5% and extracts from the stalk with leaves at ≥5% (Figure 3A, Figure 4 and Figure 5).

The growth of whole *T. repens* seedlings and their underground parts was significantly inhibited compared to that of the control by all extracts used, and the effect of the extracts increased with the increase in their concentration. The length of the aboveground parts of white clover was significantly shorter in seedlings watered with both extracts at ≥5% (Figure 3B, Figure 4 and Figure 5).

### 2.5. Fresh and Dry Masses and Total Water Content

A reduction in the fresh mass of *F. rubra* seedlings was demonstrated when watered with extracts from the ear and stalk with leaves of *H. murinum* at a concentration of 7.5%. In contrast, 2.5% ear extract stimulated an increase in its fresh mass. The extracts from the ear caused a significant reduction in the fresh mass of *T. repens* at 7.5%, and the extracts from the stalk with leaves at ≥2.5%. *H. murinum* ear extracts did not have a statistically significant effect on the dry mass of *F. rubra* seedlings, while the dry mass of *T. repens* seedlings watered with extracts at 2.5% and 5% was significantly higher than that of the control. The extracts from the stalk with leaves significantly reduced the dry mass of *F. rubra* seedlings only at 7.5%, while in *T. repens* seedlings, all the concentrations applied resulted in an increase in dry mass compared to the control. All types of extracts reduced the water content in tissues of *T. repens* but had no significant effect on the water content in tissues of *F. rubra* (Table 4).

### 2.6. Electrolyte Leakage

**Figure 6:**
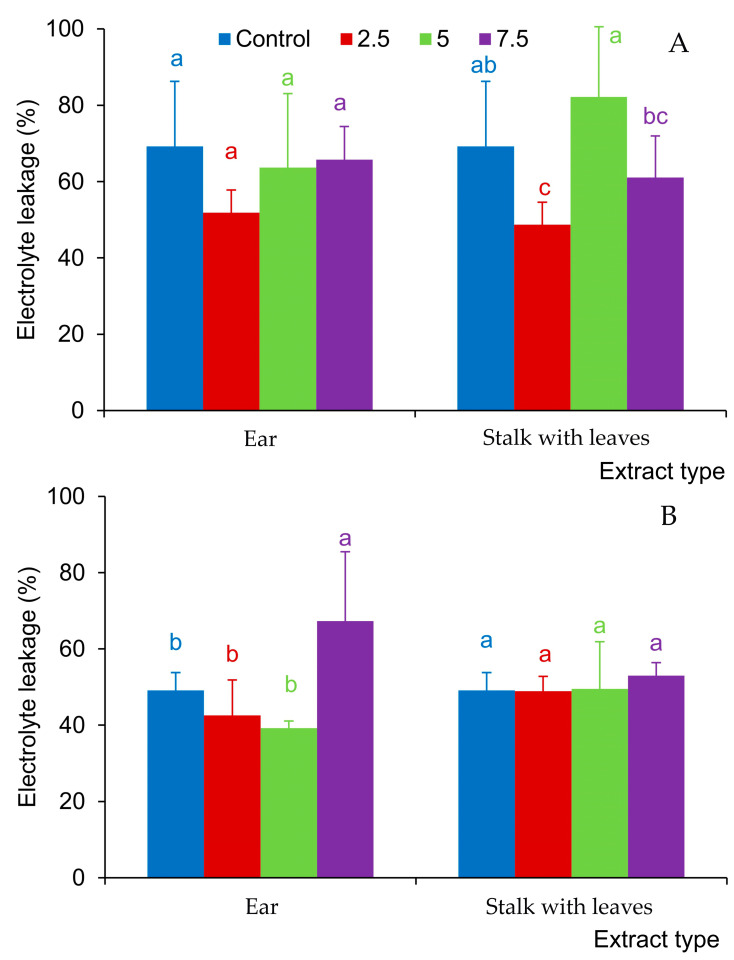
Electrolyte leakage from seedlings of *Festuca rubra* L. (**A**) and *Trifolium repens* L. cv. Grassland Huia (**B**), watered with aqueous extracts (2.5%, 5%, 7.5%) from the organs of *Hordeum murinum* L. subsp. *murinum*; mean values of 5 replicates (±SD) marked with different letters differed significantly according to Duncan’s test *p* ≤ 0.05.

The extracts from the stalk and leaves reduced the degree of destabilization of the cell membranes of the *F. rubra* seedlings at the concentration of 2.5% (Figure 4A), and in clover, they did not cause significant changes in the electrolyte leakage from the analyzed seedlings (Figure 4B). Ear extracts did not have a significant effect on electrolyte leakage in *F. rubra* seedlings (Figure 6A). In the case of white clover, they increased the electrolyte leakage in seedlings only at the concentration of 7.5% (Figure 6B).

## 3. Discussion

Plants communicate with each other and with the environment through the oldest mode of communication, which is called ‘chemical language’ [8,9]. The ‘language’ consists mainly of secondary metabolites in the form of ‘words’, estimated to be more than 400,000 [31,32]. Secondary metabolites with allelopathic effects include alkaloids, cyanogenic glycosides, terpenes, terpenoids, waxes, tannins, steroids, alcohols, phenols, aldehydes, ketones, esters, and nonprotein amino acids [6,33,34,35]. Such compounds are also found in various concentrations in *Hordeum murinum* L. subsp. *murinum,* as our study has demonstrated (Table 2 and Figure 1). These compounds create the basis of the allelopathic potential of this invasive plant, which can spread in poor anthropogenic habitats, as also is confirmed in this study (Table 1). The presence of compounds with allelopathic potential in *H. murinum* fits the novel weapon hypothesis, which explains the invasiveness of species by using compounds foreign to native plants as an effective weapon in the struggle for competing resources [14,36]. This hypothesis has received great interest and can well explain the competitive victory of many alien species (e.g., [37,38,39]). Secondary plant metabolites also play an important protective role against pathogens, excessive water loss (e.g., waxes), and oxidizing factors (terpene compounds). Our study indicated the allelopathic effects of *H. murinum* extracts on the germination and early growth stages of two common grassland species (Table 5).

The inhibitory effect of allelopathic compounds is one of the reasons for determining the possible winner in resource competition [40,41], and secondary metabolites play many ecological roles [42,43,44].

The study by Toravane and Mokat [45] found that among the allelochemicals isolated from *Neanotis montholonii* (Hook.f.) W.H.Lewis, linoelaidic acid most inhibited the germination and the growth of roots and shoots of mung beans and rice. In our experiment with *H. murinum*, among the detected allelochemicals, the highest percentage of linoelaidic acid was recorded (Table 2). Probably also in this case it is a compound that has a high allelopathic potential in relation to the tested meadow species. The second most common substance in *H. murinum* was n-hexadecanoic acid (Table 2). Its allelopathic properties were confirmed by examining the growth and development of wheat seedlings [46].

**Figure 7:**
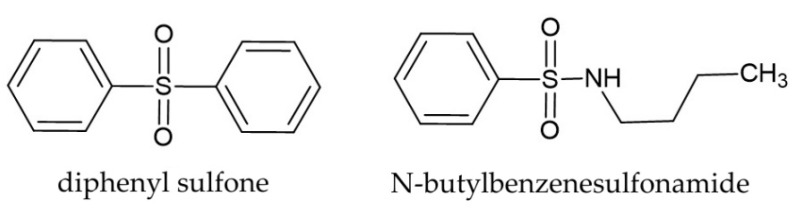
Structural formulas of diphenyl sulfone and N-butylbenzenesulfonamide.

According to Li et al. [47], for chemicals released from plants to have allelopathic effects, they need to diffuse and spread to different locations in the soil environment close to the target species. The compounds present in *H. murinum* extracts, such as ferulic acid, p-hydroxybenzoic acid, and vanillic acid, according to these authors, show poor mobility in soil, which limits their allelopathic effects on the target plants. However, their mobility can increase if they combine with other allelochemicals. For example, the combination of phenolic compounds and sulfonic compounds (e.g., dimethyl sulfoxide DMSO) has strong allelopathic properties [48]. In our study, these compounds were N-butylbenzenesulfonamide and diphenyl sulfone (Table 2; Figure 1). Both compounds were identified in other plants, e.g., diphenyl sulfone was found in *Myriactis humilis* Merr [49], and N-butylbenzenesulfonamide in *Angelica sinensis* (Oliv.) Diels [50]. However, these compounds are not commonly reported from plants. Their presence could be responsible for the increased allelopathic properties of *H. murinum*, as previously suggested for other species. In particular, these compounds (Figure 7) have not been previously documented for *H. murinum* and for the *Poaceae* family.

N-butylbenzenesulfonamide is one of the compounds naturally found in the bark extract of *Prunus africana* (Hook.f.) Kalkman. It is a sulfur-containing compound that is widely used as a plasticizer in polyacetals and polyamides and it shows high antiandrogenic activity [51]. N-butylbenzenesulfonamide inhibits the growth of microflora near plants and it has antifungal properties, ED_50_, values of the N-butylbenzenesulfonamide against *Pythium ultimum* Trow, *Pytophthora capsici* Leonian, *Rhizoctomia solani* J.G. Kühn, and *Botrytis cinerea* Pers. [52]. Moreover, it shows high antiandrogenic activity [53]. Plant extracts containing diphenyl sulfone have strong radical scavenging properties. Diphenyl sulfone is a well-known pesticide; there are very few reports on its natural occurrence, most noteworthy being in the case of plants like *Myriactis humilis* Merr. [54].

Other biologically active substances were also found in plant extracts. Cyclodecanol is the main component of essential oils from the leaves and stems of Korean *Coriandrum sativum* L. This oil has significant toxic effects against the larvae of *Aedes aegypti* L. [55]. Although in the case of the extract obtained from *H. murinum*, its share is 1.0–1.4%, its presence is worth noting. Another interesting compound found in the investigated plant material was pentadecanal. This aldehyde has very strong antibacterial activity [56].

Phenolic compounds are recognized as one of the most important secondary metabolites in the allelopathy process [57,58,59,60]. Phenolics are important mediators in plant–environment interactions [61]. Released by plants into the soil, with root exudates or from plant tissues, they delay the colonization of dead plant material by microorganisms and slow down the rate of decomposition [62,63]. In studies on the chemical composition of various plant species, e.g., *Hordeum vulgare* L., *Triticum aestivum* L., *Zea mays* L. and the genus *Trifolium* [60,61,64], the highest concentration of phenolic compounds was found in their leaves, especially phenolic acids [60]. In an experiment with *H. murinum* subsp. *murinum*, the percentage of some phenolic compounds in leaves and stems was higher than that in ears (Table 2). This confirms the already-known thesis that, in addition to the roots, leaves are an important source of allelopathic substances. Studies by other authors indicate that *H. murinum* extracts inhibit germination, a finding also confirmed in the experiment conducted (Figure 2, Table 3). For example, Puig et al. [65] showed that a 24.8% aqueous extract of *H. murinum* completely inhibited the germination of seeds of *Aster squamatus* Hieron., *Conyza bonariensis* L., and significantly reduced the germination of *Bassia scoparia* (L.) A.J. Scott. The authors found that aqueous extracts from *H. murinum* contained phenolic acids (e.g., protocatechuic, vanillic, p-hydroxybenzoic, p-hydroxybenzaldehyde, etc.) and flavonoids (e.g., apigenin, apigenin derivative ‘1’, apigenin derivative ‘2’). The most abundant compounds were apigenin derivative ‘2’ (187.84 µg-mL^−1^) and ‘1 (150.18 µg-mL^−1^). These authors concluded that the phytotoxic effect of *H. murinum* extracts on *C. bonariensis* germination was probably due to the content of syringic, protocatechuic, and vanillic acids, apigenin. For *A. squamatus*, the inhibition of germination is probably explained by the content of p-hydroxybenzoic acid, protocatechuic acid, apigenin, and syringic acid in the extracts. In contrast, the germination of *B. scoparia* is mainly inhibited by the flavonoids naringenin and apigenin contained in the extracts. The greater sensitivity of the root to allelochemicals was probably due to the contact time of this organ with the extract. During germination, the seed coat is the first to be exposed to the environment, followed by the developing roots of the seedling [66].

Hamidi et al. [67] also pointed to the inhibitory effect of the wall barley extracts. They found that barley stalk extract had stronger allelopathic properties than its root extract.

The chemical compound 6,10,14-trimethylpentadecan-2-one (TMP, hexahydrofarnesyl acetone), found in our study, is known for its allelopathic and pest control potential [68,69].

Interesting results are provided by Lucero et al. [70], who observed in a study of shrub plant co-occurrence under arid conditions a facilitative interaction of native shrubs with invasive species. This may be related to the fact that native species are sensitive to allelopathic compounds, whereas alien species are not. In this way, the novel weapon hypothesis [36] can also be reversed and recalled, where allelopathins secreted by alien species can eliminate native species and further support the colonization process in addition to other alien plant traits. Considering the compounds shown in our study, it can be concluded that some allelopaths negatively affect the growth of native plants (Table 5), thus increasing the chance of eliminating potential competitors. Similar conclusions have been drawn from studies on other invasive species (e.g., [71]).

*H. murinum* also shows morphological characteristics that allow it to dominate in urban settings. In a study on plasticity and adaptation of selected species under N and P deficiency, it was found that in *H. murinum*, a high leaf area contributed to higher fitness [72]. The cited studies also confirmed the plasticity of the species under conditions of nutrient (phosphorus and nitrogen) deficiency in the soil. Both traits are important for plant competition. Furthermore, studies on the impact of global climate change have confirmed that increased CO_2_ and CO_2_ combined with higher temperatures improved biomass and the growth of *H. murinum* [73].

## 4. Materials and Methods

### 4.1. Plant Material

Specimens of *Hordeum murinum* were obtained in 2022 from roadside sites in Gorzów Wielkopolski (52°43′51″ N, 15°14′18″ E) and Piła (53°08′51″ N, 16°43′51″ E), in western Poland. Plants were collected in the flowering stage. Specimens that visually were not infected with viruses or fungi and were undamaged were selected for the experiments. The plant material was dried in the dark at room temperature and stored for the duration of the experiment in paper bags (stalks with leaves and ears separately). The voucher specimens were deposited in the Herbarium of the Department of Botany, Institute of Biology and Earth Science, University of the National Education Commission, Kraków, Poland.

Grains of red fescue (*Festuca rubra* L.) were bought from DLF Seeds (Hladké Životice, Czech Republic) and seeds of white clover (*Trifolium repens* L. cv. Grassland Huia) were obtained from the experimental station of the University of Agriculture in Kraków (Prusy, Poland).

### 4.2. Soil Analysis

To check the content of selected macronutrients at *H. murinum* L. subsp. *murinum* sites, mixed soil samples were taken. Soil samples were collected from the top layer of the soil profile (5–20 cm) at 10 points (10 samples) and then mixed to obtain a composite sample. Under laboratory conditions, soil pH (pH in H_2_O) and N-NO_3_^−^, N-NH_4_^+^, Cl^−^ content were tested using the potentiometric method. Soil salinity (in g NaCl/dm^3^) was determined using the conductometric method [74]. The content of phosphorus (P) was determined by the colorimetric method, and that of potassium (K) and calcium (Ca) by flame photometry [75]. For the determination of magnesium (Mg), the FAAS (flame atomic absorption spectrometry) method was used [76,77].

### 4.3. Gas Chromatography Methods (GC–MS)

The chromatographic profile of compounds isolated from plant material (ears and stalks with leaves separately) shows significant differences depending on the solvent used and the polarity of the compounds intended to be determined. For the extraction of plant material, methanol shows better extraction efficiency in terms of total organic compounds than n-hexane, chloroform, or ethyl acetate [78]. For these reasons, methanol was used as a solvent in this experiment.

#### 4.3.1. Extraction and Derivatization Procedure

All solvents and reagents from various suppliers were of the highest purity needed for each application. Silylation reagents, BSTFA (Ν,O-bis(trimethylsilyl) trifluoroacetamide), and TMCS (trimethylchlorosilane) were obtained from Sigma-Aldrich (St. Louis, MO, USA). The ultrasound-assisted extraction (UAE) method was used for the extraction of organic compounds from dry plant samples. The soak time was 1 h, the extraction solvent volume was 0.10 g sample per 10 mL of methyl alcohol, the microwave power was 400 W, and the extraction time was 15 min. Before derivatization, methyl alcohol was exchanged for n-hexane. For an aliquot (50 µL) in n-hexane, approximately 50 µL of BSTFA + TMCS (98:2) was added and placed in the oven for 1 h at 60 °C. Then, the unreacted BSTFA and TMCS were evaporated under a flow of nitrogen, and the content was dissolved in n-hexane prior to analysis by GC–MS.

#### 4.3.2. Main Parameters

An Agilent 6890N GC–MS with an Agilent 5975C MS operated in electron impact mode was used for sample analysis. The capillary column was an HP-5 ms (30 m × 0.25 mm × 0.25 µm). The initial oven temperature was 50 °C, held for 1 min, then raised to 285 °C at 10 °C/min with a final isotherm of 6 min, the transfer line temperature was 285 °C. The injection port and interface temperature were both 280 °C; 1 µm of the sample was injected in the splitless mode. Helium (6.0) as the carrier gas was maintained at a constant flow rate of 1 mL/min. The ionization source temperature and the MS quadrupole temperature were set at 230 °C and 150 °C, respectively. The mass spectrometer was scanned over the 45 to 600 *m*/*z* range with an ionizing voltage of 70 eV and identification was based on a standard mass library of the National Institute of Standards and Technology (version NIST 14) for compounds extracted from samples.

### 4.4. Preparation of Extracts for Allelopathic Studies

The dried parts of *H. murinum* subsp. *murinum* (separately ears and stalks with leaves), were ground in a laboratory mill, and extracts of 2.5%, 5%, and 7.5% were prepared from them (2.5 g raw material + 97.5 mL^−1^ H_2_O = 2.5% extract). For the extraction of the chemicals, the distilled water-soaked plant material was stored for 24 h in the dark at a room temperature of 23 °C ± 2 °C. After this time, the aqueous extracts were filtered through filter paper and stored for the duration of the experiment in a refrigerator at 8 °C ± 2 °C.

The values of pH and EC [µS] of the extracts were measured and the following means (3 replicates) were received for stalks with leaves: extract 2.5%: pH 5.92 and EC 3.31; extract 5%: pH 6.05 and EC 4.87, extract 7.5%: pH 6.08 and EC 6.43; for ears: extract 2.5%: pH 5.96 and EC 3.38 (µS); extract 5%: pH 6.02 and EC 4.79, extract 7.5%: pH 6.11 and EC 6.22.

### 4.5. Seed Preparation and Germination Conditions

White clover seeds and red fescue grains (separately) were sterilized in 1% acetone solution for 1 min and then rinsed three times with distilled water. Twenty-five seeds and grains each were placed on sterile Petri dishes with three layers of blotting paper moistened with an appropriate extract of wall barly. The control consisted of seeds and grains placed in Petri dishes with blotting paper moistened only with distilled water. The seeds and grains in the Petri dishes were placed in the dark, at a room temperature of 23 °C ± 2 °C, and a relative humidity of approximately 60–70%. Every 24 h for 7 days (for clover seeds) and 14 days (for fescue grains), the number of germinated seeds and grains was checked. The experiment was carried out in three replicates for each concentration and type of barley extract and control. Based on this, the germination percentage (GP) was determined according to the formula:

(1) $$GP=\frac{\sum_{i=1}^{k}n_{i}}{N}\times 100,$$

where *n*_i_—number of seeds newly germinating on day *i*; N—total number of seeds tested and *k*—last day of germination [79].

In addition, other germination indices were determined that illustrate the influence of the analyzed solutions on the germination process of the tested plants: mean germination time—MGT [80], germination index—GI [80,81], uncertainty of germination process—U [82], time to 50% germination—T_50_ [83]. These parameters were determined according to the following formulas:

MGT [day]—mean germination time

(2) $$MGT=\frac{\sum_{i=1}^{k}n_{i}t_{i}}{\sum_{i=1}^{k}n_{i}}$$

where *k*—last day of germination, *n_i_*—number of seeds newly germinating on day *i*, *t_i_*—number of days from sowing,

GI [unit less]—germination index

(3) $$GI=\sum_{i=1}^{k}\frac{n_{i}}{t_{i}}$$

where *k*—last day of germination, *n_i_*—number of seeds newly germinating on day *i*, and *t_i_*—number of days from sowing,

U [bit]—uncertainty of germination process

(4) $$U=-\sum_{i=1}^{k}f_{i}\log_{2}f_{i}\;\mathrm{where}\;f_{i}=\frac{n_{i}}{\sum_{i=1}^{k}n_{i}}$$

where f*_i_* is the relative frequency of germination (estimated as $f_{i}=\frac{n_{i}}{\sum_{i=1}^{k}n_{i}}$), *k*—last day of germination, *n_i_*—number of seeds newly germinating on day *i*, *t_i_*—number of days from sowing,

T_50_ [day]—time to 50% germination

(5) $$T_{50}=t_{i}+\frac{\left(\frac{N}{2}-n_{i}\right)\left(t_{j}-t_{i}\right)}{\left(n_{j}-n_{i}\right)}\;\mathrm{where}\;n_{i}<\frac{N}{2}<n_{j}$$

where N—final number of germination, *n_i_*, *n_j_*—cumulative number of seeds germinated by adjacent counts at times *t_i_* and *t_j_* when $n_{i}<\frac{N}{2}<n_{j}$.

### 4.6. Measurements of Elongation Growth of Studied Seedlings

Biometric measurements of white clover and fescue seedlings grown on different extracts of *H. murinum* organs were carried out by analyzing the length of the underground and aboveground parts of the seedlings using a caliper (Topex 31C615, Warszawa, Poland), with an accuracy of 0.1 mm. Measurements were performed for each concentration and organ as well as for the control sample in 10 replicates.

### 4.7. Measurements of Fresh and Dry Mass and Total Water Content

After 7 days of germination of white clover seeds and 14 days of germination of fescue grains, fresh and dry masses were determined for individual seedlings on a laboratory balance (WPS-120, Radwag, Radom, Poland). For dry mass determination, single seedlings were dried for 48 h at 105 °C in a dryer (SUP 100, WAMED, Warszawa, Poland). The calculation of the percentage water content was performed according to the following formula:

H_2_O (%) = 100 − [(DM × 100)/FM] (6)

where DM—dry mass, FM—fresh mass [84].

Measurements were performed for each concentration and organ as well as for the control sample in 5 replicates.

### 4.8. Electrolyte Leakage

The percentage of electrolyte leakage, illustrating the degree of the destabilization of the cell membranes of the white clover and fescue seedlings, was measured according to the method described in the study by Szafraniec et al. [85]. This parameter was measured on the 7th (*Trifolium repens*) and 14th day (*Festuca rubra*- later sprouting) of this experiment. Individual seedlings were placed in polypropylene falcon tubes with 30 mL of distilled water (with conductivity 0.05 µS) and shaken for 3 h (Labnet, Rocker, USA). Electrolyte leakage from living cells (E_1_) was measured using a CX-701 conductivity meter (Elmetron, Zabrze, Poland) with the electrode (K = 1.02). The falcons with seedlings in distilled water were frozen for 24 h at −25 °C to macerate the cells. After that, samples were thawed and subjected to the same shaking and measurement procedure as samples with living seedlings to determine total electrolyte leakage (E_2_). The percentage of electrolyte leakage was calculated according to the following formula:

EL (%) = (E_1_/E_2_) × 100 (7)

where EL—electrolyte leakage, E_1_—EL live seedling, E_2_—EL dead seedling.

Measurements were performed for each concentration and organ as well as for the control sample in 5 replicates.

### 4.9. Statistical Analysis

Statistica 13.3 software was used for statistical calculations. To determine the differences between the experimental values obtained, the standard deviation (±SD) was calculated for each parameter and Duncan’s test for *n* = 5 (for seedling length *n* = 10), at *p* ≤ 0.05, was applied.

## 5. Conclusions

The biological characteristics of wall barley typify this species a winner in the competition for various environmental resources, which also make it highly invasive in many parts of the world. Previous studies on this species have shown it has relatively low habitat requirements, high viability of both organism and seeds, and high trait plasticity under different environmental conditions. These traits are particularly useful in urban settings. The present study indicated that compounds produced by the plant, particularly secondary metabolites, may also play an important role in the invasiveness of this species by limiting the germination and early growth stages of meadow plants. (i) The production of two compounds of the species, N-butylbenzenesulfonamide and diphenyl sulfone, was demonstrated for the first time. The two identified compounds are found extremely rarely in nature and their biological properties are multidirectional and very unique. (ii) The results of this study indicate the need to remove the biomass of *Hordeum murinum* when attempting to restore of habitats occupied by this invasive species. Leaving the biomass can lead to the release of allelopathic compounds and inhibit the germination and growth of meadow plants.

## Figures and Tables

**Table 1:** Basic soil parameters at sites dominated by *Hordeum murinum* L. subsp. *murinum* (Gorzów, Poland).

pH in H_2_O	NaCl [g/dm^3^]	Content of Available Nutrients [mg/dm^3^ of Soil]						
		N-NO_3_ Nitrate	N-NH_4_ Ammonium	P	K	Ca	Mg	Cl
7.21	0.40	14	46	140	250	2500	142	28

**Table 2:** Chemical composition of methanolic extracts from *Hordeum murinum* L. subsp. *murinum*; the highest values are highlighted in grey.

No.	Compound	Ear—Relative Percentage [%]	Retention Time [min]	Stalk with Leaves—Relative Percentage [%]	Retention Time [min]
	Aldehydes:				
1.	5-Hydroxyfurfural	**1.0**	9.26	0.5	9.24
2.	Pentadecanal	0.5	15.38	0.4	15.36
	Alkanes and alkenes:				
3.	Hexadecane	0.8	11.56	0.4	11.56
4.	Nonadecane	0.8	14.03	<0.1	14.01
5.	Neophytadiene	4.6	16.70	**5.1**	16.70
6.	Heneicosane	1.3	19.24	0.2	19.24
7.	Tricosane	**2.1**	21.02	0.6	21.02
8.	Tetracosene	3.6	25.90	3.9	25.92
9.	Stigmasta-3,5-diene	0.6	26.59	0.5	26.59
10.	Squalene	<0.1	25.31	1.2	25.31
	Carboxylic acids and lactones:				
11.	Benzoic acid	**1.0**	8.26	0.2	8.26
12.	n-Tetradecanoic acid	**1.1**	15.84	0.5	15.84
13.	n-Hexadecanoic acid	**22.7**	19.93	18.8	19.99
14.	Linoelaidic acid	**38.1**	19.62	25.6	19.67
15.	(R)-5,6,7,7a-Tetrahydro-4,4,7a-trimethyl-2(4H)-benzofuranone	<0.1	13.39	0.4	13.39
16.	6-Hydroxy-4,4,7a-trimethyl-5,6,7,7a-tetrahydrobenzofuran-2(4H)-one	<0.1	16.08	0.4	16.08
	Phenols and alcohols:				
17.	Vanillin	0.5	11.66	0.4	11.66
18.	2,4-Ditertbutylphenol	**1.6**	13.01	0.4	13.03
19.	Cyclodecanol	1.4	15.15	1.0	15.15
20.	3,7,11,15-Tetramethyl-2-hexadecen-1-ol	1.6	16.97	1.6	16.97
21.	1-Hexacosanol	1.0	26.40	**7.3**	26.40
22.	Butylated hydroxytoluene	<0.1	14.00	0.4	14.04
23.	Phytol	<0.1	19.20	**1.8**	19.24
24.	Behenic alcohol	3.6	25.92	3.9	25.92
	Sterols:				
25.	Campesterol	1.8	31.40	**4.4**	31.40
26.	γ-Sitosterol	4.6	33.32	**8.2**	33.35
27.	β-Sitosterol	3.3	32.54	**7.8**	32.58
28.	3-β-Cholestan-4-en-3-ol	<0.1	29.50	0.6	29.50
29.	3-β-Ergost-5-en-3-ol	1.0	30.75	**2.4**	30.75
	Others:				
30.	N-butylbenzenesulfonamide (NBBS)	**0.8**	16.21	0.2	16.21
31.	Diphenyl sulfone (DDS)	0.5	17.73	<0.1	17.73
32.	6,10,14-Trimethylpentadecan-2-one (TMP)	<0.1	17.79	1.1	17.79

**Table 3:** Germination rates of *Trifolium repens* L. cv. Grassland Huia (A) and *Festuca rubra* (B) seeds watered with extracts from the organs of *Hordeum murinum* L. subsp. *murinum* with different percentage concentrations (2.5%, 5%, 7.5%). MGT—mean germination time (days), U—uncertainty of germination process, GI—germination index, T_50_—time to 50% germination. Means marked with different letters differed significantly according to Duncan’s test *p* ≤ 0.05.

Extract Type (%)	MGT		U		GI		T_50_	
	A	B	A	B	A	B	A	B
	Ear							
Control	2.27 b ±0.38	10.93 b ±0.12	1.71 a ±0.56	3.05 a ±0.04	13.64 a ±3.80	8.92 a ±0.68	1.69 b ±0.15	10.63 b ±0.07
2.5	2.96 a ±0.13	11.02 b ±0.30	1.88 a ±0.15	2.97 ab ±0.12	6.98 b ±0.39	6.65 ab ±2.35	2.34 a ±0.17	10.68 b ±0.26
5	3.02 a ±0.47	11.26 ab ±0.25	1.80 a ±0.04	2.92 ab ±0.11	6.55 b ±1.32	5.83 b ±0.60	2.31 a 0.17	10.98 ab ±0.30
7.5	2.76 ab ±0.15	11.75 a ±0.52	1.59 a ±0.21	2.67 b ±0.29	4.64 b ±1.70	2.31 c ±0.87	2.16 a ±0.20	11.47 a 0.45
	Stalk with leaves							
Control	2.27 c ±0.38	10.93 b ±0.12	1.71 a ±0.56	3.05 a ±0.04	13.64 a ±3.80	8.92 a ±0.68	1.69 c ±0.15	10.63 b ±0.07
2.5	2.64 bc ±0.20	11.00 b ±0.16	1.93 a ±0.16	3.01 a ±0.04	7.96 b ±0.76	7.10 ab ±1.80	2.14 bc ±0.40	10.67 b ±0.18
5	3.02 ab ±0.38	11.18 ab ±0.48	1.71 a ±0.48	2.96 ab ±0.15	6.24 b ±0.20	5.92 b ±1.96	2.53 ab ±0.24	10.94 ab ±0.54
7.5	3.46 a ±0.47	11.87 a ±0.66	1.70 a 0.34	2.62 b ±0.35	3.99 b ±2.04	2.28 c ±0.84	2.91 a ±0.25	11.65 a ±0.64

**Table 4:** Fresh and dry mass and water content in seedlings of *Festuca rubra* L. (A) and *Trifolium repens* L. cv. Grassland Huia (B), watered with aqueous extracts (2.5%, 5%, 7.5%) from the organs of *Hordeum murinum* L. subsp. *murinum*; stimulation is highlighted in gray; the mean values given of 5 replicates (±SD) marked with different letters differed significantly according to Duncan’s test *p* ≤ 0.05.

Extract Type (%)	Fresh Mass (g)		Dry Mass (g)		Total Water Content (%)	
	A	B	A	B	A	B
Ear						
Control	0.0059 b ±0.0010	0.0059 a ±0.0000	0.0010 ab ±0.0001	0.0003 c ±0.0000	83.1933 a ±3.2783	94.5942 a ±0.2602
2.5	0.0073 a ±0.0013	0.0057 a ±0.0013	0.0010 a ±0.0002	0.0005 ab ±0.0001	85.8796 a ±2.8015	91.9138 b ±1.0159
5	0.0059 b ±0.0004	0.0053 ab ±0.0009	0.0008 ab ±0.0001	0.0005 a ±0.0001	85.7143 a ±2.0797	89.9071 c ±2.8838
7.5	0.0043 c ±0.0007	0.0046 b ±0.0006	0.0007 b ±0.0002	0.0004 bc ±0.0001	83.3605 a ±3.1469	91.5094 bc ±0.5973
Stalk with leaves						
Control	0.0059 a ±0.0010	0.0059 a ±0.0000	0.0010 a ±0.0001	0.0003 b ±0.0000	83.1933 a ±3.2783	94.5942 a ±0.2602
2.5	0.00572 a ±0.0005	0.0052 b ±0.0012	0.0010 a ±0.0001	0.0006 a ±0.0001	82.3205 a ±3.0180	88.5846 b ±1.3340
5	0.00492 a ±0.0005	0.0045 c ±0.0020	0.00082 ab ±0.0001	0.0006 a ±0.0001	83.3782 a ±1.6657	85.5712 c ±4.1885
7.5	0.00244 b ±0.0009	0.0043 c ±0.0002	0.00064 b ±0.0003	0.0006 a ±0.0000	69.3282 a ±19.1781	86.1026 c ±0.2433

**Table 5:** Comparison of the germination and growth parameters of *Festuca rubra* L. and *Trifolium repens* L. cv. Grassland Huia seedlings grown on the aqueous extracts from the organs of *Hordeum murinum* L. subsp. *murinum*, with different percentage concentrations (2.5%, 5%, 7.5%).

	Extracts	*Festuca rubra*						*Trifolium repens* cv. Grassland Huia					
		Ear			Stalk with Leaves			Ear			Stalk with Leaves		
Parameter		2.5%	5%	7.5%	2.5%	5%	7.5%	2.5%	5%	7.5%	2.5%	5%	7.5%
GP													
MGT													
U													
GI													
T_50_													
Length (whole seedling)													
FM													
DM													
TWC													
EL													

No differences relative to the control—blue, positive effect relative to the control—green, negative effect relative to the control—red; GP—germination percentage, MGT—mean germination time, U—uncertainty of germination process, GI—germination index, T_50_—time to 50% germination, FM—fresh mass, DM—dry mass, TWC—total water content, EL—electrolyte leakage.

## Data Availability

Data are contained within the article.
